# Lymphatic endothelial cells - key players in regulation of tolerance and immunity

**DOI:** 10.3389/fimmu.2012.00305

**Published:** 2012-09-28

**Authors:** Eric F. Tewalt, Jarish N. Cohen, Sherin J. Rouhani, Victor H. Engelhard

**Affiliations:** ^1^Department of Microbiology, Immunology, and Cancer Biology, University of Virginia School of MedicineCharlottesville, VA, USA; ^2^Carter Immunology Center, University of Virginia School of MedicineCharlottesville, VA, USA

**Keywords:** lymphatic endothelial cells, tolerance, trafficking, inflammation, antigen presentation

## Abstract

The lymphatic vasculature provides routes for dendritic cell and lymphocyte migration into and out of lymph nodes. Lymphatic endothelial cells (LEC) control these processes by expression of CCL21, sphingosine-1-phosphate, and adhesion molecules. LEC express MHC-I and MHC-II, but not costimulatory molecules, and present antigen on MHC-I via both direct and cross-presentation. Whether LEC present to CD4 T cells on MHC-II is unknown. Interestingly, LEC express antigens otherwise restricted to a small number of peripheral tissues in an autoimmune regulatory element-independent manner. Direct presentation of peripheral tissue antigens (PTA) to CD8 T cells results in abortive proliferation and deletion, due to both a lack of costimulation and active PD-L1 engagement. Autoimmunity develops when deletion is subverted, suggesting that LEC presentation of PTA could lead to human disease if PD-1 signaling were impaired by genetic polymorphisms, or aberrant costimulation occurred during inflammation. The expression of additional inhibitory molecules, which are not involved in LEC-mediated deletion, suggests that LEC may have additional immunoregulatory roles. LEC express receptors for several immunomodulatory molecules whose engagement alters their phenotype and function. In this review we describe the role of LEC in distinct anatomical locations in controlling immune cell trafficking, as well as their emerging role in the regulation of T cell tolerance and immunity.

## LYMPHATIC ENDOTHELIAL CELLS REGULATE THE TRAFFICKING OF DENDRITIC CELLS AND LYMPHOCYTES BETWEEN TISSUES AND SECONDARY LYMPHOID ORGANS

Lymphatic endothelial cells (LEC) compose the lymphatic vasculature, which maintains tissue fluid balance and transports antigen and dendritic cells (DC) to the lymph node (LN). Lymphatic vasculature in tissues is composed of blind-ended capillary-like structures, termed initial lymphatics (Leak, 1976), which join to form larger collecting lymphatic vessels (Schmid-Schönbein, 1990) and ultimately feed into the LN subcapsular sinus. Within the LN, LEC are localized to the subcapsular, cortical, and medullary sinuses, where they interact with incoming and exiting leukocytes (Grigorova et al., 2010).

Whereas the blood vasculature in peripheral tissues attracts leukocytes to inflamed sites to exert effector functions, the lymphatic vasculature facilitates the induction of immunity and tolerance. DC enter the initial lymphatics through portals in the basement membrane (Lämmermann et al., 2008; Pflicke and Sixt, 2009). T cells are likely to enter in a similar manner. LEC of the initial lymphatics express CCL21-Leu, one of two CCL21 isoforms in mice, in punctate clusters on the abluminal surface (Vassileva et al., 1999; Tal et al., 2011). CCL21-Leu is the primary determinant for DC entry through engagement of CCR7, but it is not expressed by LEC in LN and does not mediate migration to the node itself (Vassileva et al., 1999; Luther et al., 2000; Nakano and Gunn, 2001). Humans express a single CCL21 isoform, which encompasses the functions of both murine isoforms. LEC that form dermal lymphatics also express CXCL12, which mediates DC entry via CXCR4 (Kabashima et al., 2007).

Extravasation of lymphocytes from blood vasculature is highly integrin dependent; however, the requirement for integrin-mediated entry into the initial lymphatics is controversial. Although LEC in the initial lymphatics express ICAM-1, and engagement of immobilized CCL21 promotes DC integrin activation and adhesion to ICAM-1 *in vitro* (Schumann et al., 2010), steady-state migration of DC into LN *in vivo* does not require integrin engagement (Lämmermann et al., 2008). This suggested other adhesion molecules may be involved. Recently, it was discovered that DC migration into lymphatic vessels and into the T cell zone of the LN requires CLEC-2 binding to podoplanin, a glycoprotein expressed by lymphatic vessel and LN-LEC as well as fibroblastic reticular cells (FRC; Acton et al., 2012). Other potential candidates include the scavenger receptor CLEVER-1, which has been implicated in the transmigration of T cells into the lumen of initial lymphatic vessels (Salmi et al., 2004). Thus, LEC-mediated entry into the afferent lymphatics is distinct from blood vascular endothelium-mediated entry of leukocytes into tissues.

Lymphatic endothelial cells also mediate the migration of DC into the LN. Once inside the collecting vessels, DC, and presumably T cells, detach from LEC and rhythmic vessel contractions propel DC toward the LN (Randolph et al., 2005). LN-LEC as well as FRC make CCL19 and CCL21-Ser, which mediate direct entry into the LN (Vassileva et al., 1999; Luther et al., 2000; Nakano and Gunn, 2001). It has been hypothesized that LEC in the collecting lymphatics also make these chemokines (Randolph et al., 2005). Additionally, LEC in the subcapsular sinus express CCL1, which can facilitate cell entry into LN (Qu et al., 2004; Kabashima et al., 2007). Once in the subcapsular sinus, DC enter the LN cortex immediately, while T cells enter the LN paracortex via medullary lymphatic sinuses (Braun et al., 2011). It is unclear how these different routes of entry are regulated. We have found that medullary and subcapsular LEC differentially express MAdCAM-1 (unpublished). These results suggest that cellular trafficking into and through the lymphatics is based on anatomically and molecularly distinct subpopulations of LEC that have different functional properties.

Lymphatic endothelial cells also control egress of lymphocytes from the LN. Upon LN entry, lymphocytes downregulate CCR7 and exit the LN through cortical and/or medullary lymphatic sinuses (Britschgi et al., 2008; Pham et al., 2008). Lymphocytes adhere to LEC and probe the sinus lumen prior to exit. Candidate molecules for adherence include CLCA1 (Furuya et al., 2010) and mannose receptor (MR; Irjala et al., 2001). Both of these molecules are more highly expressed on LN-LEC than tissue LEC (Irjala et al., 2001; unpublished). The binding partners for CLCA1 include LFA-1 and MAC-1, and *in vitro* studies demonstrated a greater role for CLCA1–LFA-1 interactions than ICAM-1–LFA-1 interactions in lymphocyte adhesion to LEC cell lines (Furuya et al., 2010). Also, MR binds CD62L, and blockade of MR on frozen LN-sections decreased lymphocyte adherence to LEC (Irjala et al., 2001). LEC are also the sole producers of sphingosine-1-phosphate (S1P) in the LN which promotes lymphocyte egress by binding to S1P1 (Pham et al., 2008; Cyster and Schwab, 2012). Lymphocyte migration and adherence to LEC, as well as probing of the cortical sinus lumen, is S1P independent. Instead, S1P is necessary for commitment to lumen entry (Grigorova et al., 2009). Thus, although the role for LEC S1P in mediating lymphocyte egress is well established, the importance of CLCA1 and MR in mediating lymphocyte egress *in vivo* remains to be examined. Collectively, these results establish the importance of chemokine and sphingolipid ligands released by LEC in controlling all phases of DC and/or lymphocyte migration in and around LN. However, the involvement of integrins or other molecules that could mediate additional adhesive interactions remains to be clarified.

## LN-LEC FUNCTION AS SPECIALIZED ANTIGEN PRESENTING CELLS

Lymphatic endothelial cells share a number of characteristics with professional antigen presenting cells (APC). LEC in LN, but not those in tissue lymphatics, constitutively express MHC-II molecules (Amatschek et al., 2007; Tripp et al., 2008; unpublished), suggesting there is a functional immunological difference between LEC in these two locations. LEC also endocytose and cross-present MHC-I antigens, although they do so less efficiently than professional APC (Lund et al., 2012). However, LEC do not express costimulatory molecules CD80, CD86, 4-1BBL, or OX40L extracellularly or intracellularly (Tewalt et al., 2012). LEC express CD70 intracellularly but it is unknown whether this is functional. LEC also express ICAM-1 and LFA-3, and LFA-3 can provide costimulation to enhance IL-2 secretion from activated T cells *in vitro* (Nörder et al., 2012). The lack of costimulatory molecule expression on LEC predisposes them to induce tolerance (see below). While professional APC upregulate costimulatory molecules upon toll-like receptor (TLR) stimulation, TLR3 ligation does not substantially upregulate CD80 or CD86 expression on LEC (Fletcher et al., 2010). It is unknown whether LEC can upregulate costimulatory molecules under other inflammatory conditions, enabling them to induce immunogenic responses. Thus, steady-state LN-LEC are semi-professional APC: they express MHC-II, activate naïve T cells and cross-present antigen, but do not constitutively express costimulatory molecules and are not known to induce outcomes other than tolerance.

Although LN-LEC express MHC-II molecules, there is limited information about the functionality of the class II processing pathway and antigen presentation to CD4 T cells. Peptide-pulsed LEC induce proliferation of naïve CD4 cells, indicating the MHC-II molecules are functional (unpublished). LEC endocytose and process exogenous antigens leading to cross-presentation on MHC-I (Lund et al., 2012), but it is not known whether this also leads to presentation on MHC-II. However, cultured human LEC do not induce allogeneic proliferation of CD4 T cells (Nörder et al., 2012). In mice that selectively express β-galactosidase (β-gal) in LEC and FRC, adoptively transferred β-gal specific CD4 T cells proliferate (Onder et al., 2011), but it was not determined whether this was due to direct antigen presentation by LEC and/or FRC, or to antigen endocytosis and presentation by hematopoietic cells. We have found that in mice expressing β-gal under control of the LEC-specific Lyve-1 promoter, proliferation of β-gal specific CD4 cells is due to presentation by hematopoietic cells (unpublished). Thus, LEC can provide antigens to DC for MHC-II presentation, analogous to medullary thymic epithelial cell (mTEC) handoff of antigens to thymic DCs (Koble and Kyewski, 2009). Further work will elucidate whether the failure of LEC to induce CD4 proliferation is due to a defect in MHC-II processing and presentation, active suppression by regulatory T cells or by molecules such as IDO or nitric oxide (NO), or induction of anergy.

## LEC AND PTA EXPRESSION

Recently, we and others have shown that multiple subsets of LN stromal cells (LNSC), including LEC, express peripheral tissue antigens (PTA) that are otherwise restricted to one or a few tissues such as skin, pancreas, gut, and central nervous system (Lee et al., 2007; Nichols et al., 2007; Gardner et al., 2008; Cohen et al., 2010; Fletcher et al., 2010). Microarray analysis comparing LN-LEC and LN blood endothelial cells (BEC) identified several additional candidates for LEC-expressed PTA (unpublished). The majority of these PTA were overexpressed in LN-LEC compared to tissue LEC, suggesting the LN microenvironment plays a role in determining PTA expression. Presentation of epitopes derived from PTA by LEC, FRC, and extrathymic autoimmune regulatory element (Aire) expressing cells (eTAC) leads to CD8 T cell abortive proliferation and deletion (Lee et al., 2007; Nichols et al., 2007; Gardner et al., 2008; Cohen et al., 2010; Fletcher et al., 2010). Collectively, these findings suggest that LN-LEC and other PTA-expressing LNSC perform a function in the periphery analogous to that of mTEC in the thymus in promoting systemic tolerance.

The molecular mechanisms controlling PTA expression by LEC have not yet been established. PTA expression by LEC is not dependent on the Aire, which controls PTA in mTEC and eTAC (Anderson et al., 2002; Gardner et al., 2008; Cohen et al., 2010). One LEC-expressed PTA, Ppy, is regulated by Deaf-1, a member of the SAND transcription factor family that includes Sp100, Aire, and NucP41/75 (Yip et al., 2009). However, Deaf1 has not been shown to regulate other LEC-expressed PTA. Deaf1 and other SAND family members are expressed at comparable levels in all LNSC subsets (Fletcher et al., 2010; unpublished), so it is unclear how Deaf1 would regulate the expression of non-overlapping PTA in different LNSC populations. However, it is also unknown how Aire controls distinct PTA repertoires in mTEC and eTAC. It is possible that the control of non-overlapping PTA repertoires in different cells by the same transcriptional regulator is due to differences in chromosomal positioning and/or epigenetic modifications. Another possibility is that multiple transcription factors play a role in LNSC PTA expression.

## CONSEQUENCES OF CD8 ANTIGEN PRESENTATION BY LEC

**FIGURE 1:**
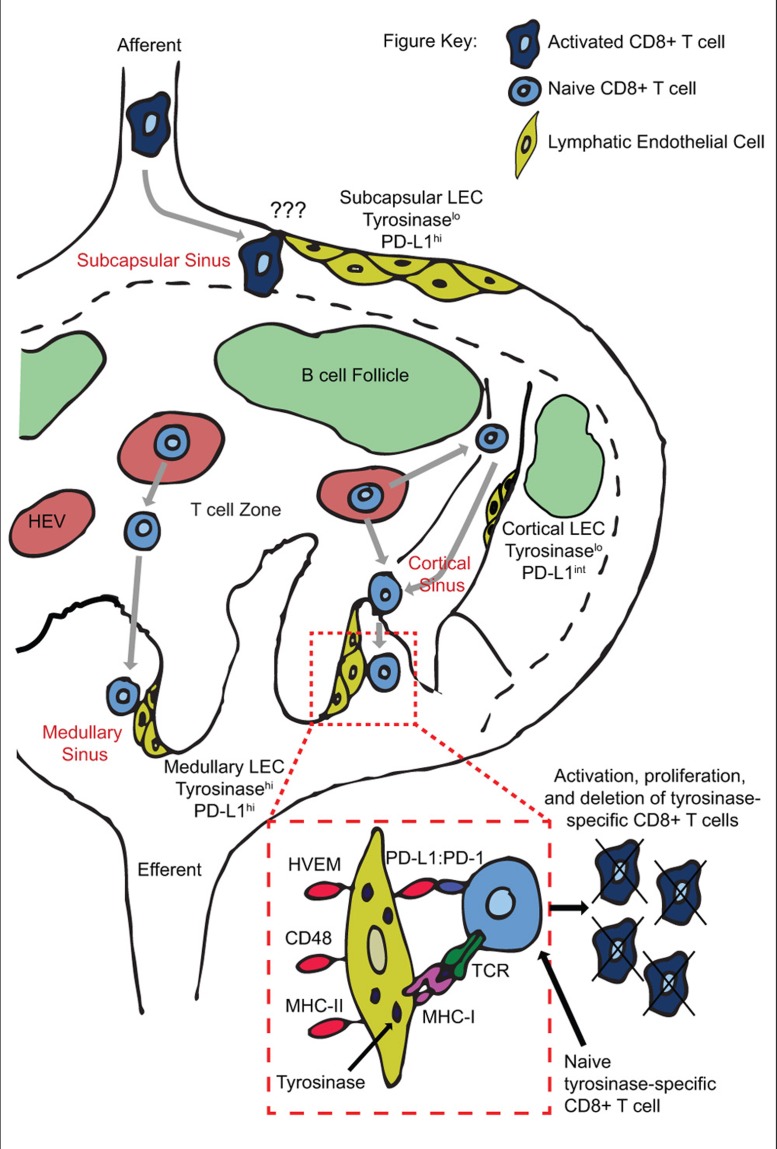
**Peripheral tolerance induction by anatomically distinct subsets of lymphatic endothelial cells during T cell trafficking through lymph nodes.** Naïve T cells enter the LN through high endothelial venules, and exit via cortical and/or medullary sinuses. Tyrosinase presentation occurs on medullary but not cortical sinus LEC, leading to proliferation and PD-L1 mediated deletion of tyrosinase-specific T cells. Deletion may occur based on engagement of PD-L1^hi^ medullary sinus LEC in the same LN as activation occurs, and/or on PD-L1^hi^ subcapsular sinus LEC in downstream LN.

As mentioned above, despite sharing some characteristics with professional APC, antigen presentation by LEC leads to tolerance. Direct presentation of tyrosinase by LN-LEC induces abortive proliferation and deletion of tyrosinase-specific T cells *in vivo* (Nichols et al., 2007; Cohen et al., 2010; **Figure 1**). Utilizing β-gal driven under control of the LEC specific Lyve-1 promoter, LEC also induce abortive proliferation and deletion of β-gal specific CD8 T cells (unpublished). Presentation of exogenous antigen by LEC was also shown to induce CD8 apoptosis *in vitro* (Lund et al., 2012). In other models, antigen level determines whether CD8 T cells undergo anergy or deletion (Redmond et al., 2005). It remains to be clarified whether LEC can induce outcomes other than deletion.

We have recently elucidated the mechanism by which LEC induce abortive proliferation and deletion of PTA-specific CD8 T cells (Tewalt et al., 2012). LEC-mediated deletion requires both a lack of costimulation and signaling through the PD-L1:PD-1 pathway (**Figure 1**). Lack of costimulation leads to rapid and elevated expression of PD-1 on T cells. Signaling through PD-1 blocks upregulation of IL-2R, which is at least in part responsible for apoptotic death (Tewalt et al., 2012). PD-1 signaling had previously only been associated with downregulation of IL-2 itself (Carter et al., 2002; Chikuma et al., 2009). These results integrate previous demonstrations that tolerance is due either to a lack of costimulation (Harding et al., 1992; Hawiger et al., 2001; Hernandez et al., 2002) or to engagement of inhibitory molecules (Martin-Orozco et al., 2006; Nurieva et al., 2006; Goldberg et al., 2007; Tsushima et al., 2007; Liu et al., 2009; Reynoso et al., 2009), and shows that they are actually interdependent pathways. Importantly, antigen presentation by LEC leads to the development of autoimmune disease when PD-L1 is blocked or exogenous costimulation is provided (Tewalt et al., 2012). Based on previous findings that LEC express multiple PTA (Cohen et al., 2010; Fletcher et al., 2010), this opens the possibility that dysregulation of their tolerance inducing capability might influence the development of some human autoimmune diseases. Finally, LEC express ligands for additional inhibitory pathways, including HVEM:BTLA/CD160, MHC-II:LAG-3, and CD48:2B4. These pathways are known to induce additional forms of tolerance, including anergy and Treg formation (Huang et al., 2004; Grosso et al., 2007; Liu et al., 2009). They are not involved in LEC-mediated abortive proliferation and deletion, but their expression suggests that LEC may have additional immunoregulatory roles under steady-state conditions.

We have also investigated the anatomical basis of CD8 abortive proliferation and deletion. LN-LEC express higher levels of PD-L1 than other LNSC populations or tissue lymphatic LEC (Tewalt et al., 2012; unpublished). The low level of PD-L1 and PTA expression by tissue LEC suggests that they are unlikely to induce tolerance. In addition, medullary and subcapsular sinus LEC express higher levels of PD-L1 than those in the cortical sinus. Importantly, tyrosinase epitope presentation to CD8 T cells is confined to medullary sinus LEC, consistent with their higher expression of tyrosinase message (unpublished; **Figure 1**). This suggests that abortive proliferation and deletion occurs as T cells attempt to exit the LN. Whether the lower level of PD-L1 expressed by cortical sinus LEC is also capable of inducing tolerance to antigens expressed at a higher level than that of tyrosinase remains to be examined. However, the high-level expression of PD-L1 by medullary sinus LEC suggests they may also induce the deletion of egressing T cells activated by other LN-resident tolerogenic APC that express low levels of PD-L1, such as FRC.

## OTHER FACETS OF IMMUNE CROSS-TALK BETWEEN LEC AND LEUKOCYTES

Lymphatic endothelial cells express multiple TLR, as well as receptors for inflammatory cytokines (Link et al., 2007; Pegu et al., 2008; Kataru et al., 2011), which enable them to respond to changes in tissue and LN microenvironments. Stimulation of cultured tissue or LN-LEC with TLR agonists, TNFα, IL-1, or infection with cytomegalovirus induces the expression of numerous chemokines (Pegu et al., 2008; Sawa et al., 2008a,b; Fiorentini et al., 2011; Garrafa et al., 2011), but the role of this enhanced expression *in vivo* has not been established. In contrast to the steady-state, DC entry into LN under inflammatory conditions is dependent upon ICAM-1 and VCAM-1, which are also upregulated on LEC by proinflammatory agents (Johnson et al., 2006; Pegu et al., 2008; Sawa et al., 2008a,b; Fiorentini et al., 2011; Garrafa et al., 2011). Inflammation also leads to proliferation and sprouting of LEC, a process known as lymphangiogenesis, by inducing the production of ligands for VegfR2, VegfR3, and the lymphotoxin β receptor (LtβR; Angeli et al., 2006; Furtado et al., 2007; Kim et al., 2009; Flister et al., 2010; Mounzer et al., 2010). Lymphangiogenesis following skin inflammation aids in the resolution of inflammation by increasing lymph flow and cell migration to the draining LN, but lymphangiogenesis following peritoneal inflammation reduces lymphatic drainage (Kataru et al., 2009; Kim et al., 2009). LN lymphangiogenesis has been shown to promote lymphocyte egress during prolonged inflammation (Tan et al., 2012). This suggests that one of the primary functions of LEC exposed to inflammatory agents is to attract a range of innate and adaptive immune cells into lymphatics to broaden and sustain ongoing immune responses.

In addition to enhancing leukocyte migration during inflammation, LEC attenuate T cell responses. TNFα activated LEC downregulate CD86 on DC, impairing their ability to induce T cell proliferation (Podgrabinska et al., 2009). LEC also limit T cell proliferation (Khan et al., 2011; Lukacs-Kornek et al., 2011) through release of NO in response to IFNγ and TNFα (Lukacs-Kornek et al., 2011). However, T cells undergoing LEC-mediated abortive proliferation and deletion produce little to no IFNγ and TNFα (unpublished). Thus, NO is unlikely to participate in LEC-mediated peripheral tolerance, but may limit the size of an immune response. Cortical sinus LEC, which express an intermediate level of PD-L1, upregulate PD-L1 in response to TLR3 ligation and IFNγ to match the high levels seen on medullary and subcapsular sinus LEC (unpublished). This could broaden the anatomical locations in the LN in which T cell tolerance occurs, or provide a means to protect cortical sinus LEC from being destroyed by emigrating effector T cells.

Inflammation modulates the expression of PTA in LEC, but not in a consistent manner. TLR3 ligation causes LEC, as well as FRC, to downregulate proteolipid protein but upregulate α-fetoprotein (Fletcher et al., 2010). However, double-negative LNSC upregulated both PTA, while BEC upregulated only one. The effect of inflammatory signals on other PTA expressed in LEC has not been examined. Downregulation of PTA could provide a means to avoid the induction of autoimmunity resulting from the increased availability of costimulation in an inflamed LN. Conversely, upregulation of PTA, particularly in the context of enhanced expression of PD-L1, could provide a means to enforce tolerance more stringently.

## CONCLUDING REMARKS

Recent work has conclusively demonstrated that LEC play a variety of active roles in shaping immune responses and tolerance. LEC guide lymphocyte and DC trafficking into and out of the LN, and inflammation increases their ability to attract cells. LEC also actively enforce CD8 T cell tolerance to PTA through their high-level expression of PD-L1 and lack of costimulatory molecules. It will be immensely interesting to determine the ways in which other inhibitory molecules expressed by LEC control T cell fate. In addition, the general immunoregulatory role of LEC will be more definitively established by understanding their ability to directly induce CD4 tolerance or to serve as a reservoir of PTA for presentation by DC. Furthermore, the identification of a second transcriptional control mechanism, in addition to Aire, will provide the possibility to understand the basis for additional human autoimmune diseases. Finally, LEC represent attractive therapeutic targets to control autoimmunity and prevent transplant rejection or to enhance tumor immuno- therapy.

## Conflict of Interest Statement

The authors declare that the research was conducted in the absence of any commercial or financial relationships that could be construed as a potential conflict of interest.
